# A Pharmaco-Metabonomic Study on Chronic Kidney Disease and Therapeutic Effect of Ergone by UPLC-QTOF/HDMS

**DOI:** 10.1371/journal.pone.0115467

**Published:** 2014-12-23

**Authors:** Ying-Yong Zhao, Hua Chen, Ting Tian, Dan-Qian Chen, Xu Bai, Feng Wei

**Affiliations:** 1 Key Laboratory of Resource Biology and Biotechnology in Western China, Ministry of Education, The College of Life Sciences, Northwest University, Xi'an, China; 2 National Institutes for Food and Drug Control, State Food and Drug Administration, Beijing, China; 3 Solution Centre, Waters Technologies (Shanghai) Ltd., Shanghai, China; Mayo Clinic, United States of America

## Abstract

Chronic kidney disease (CKD) is an important public health problem. Ergone has been proved to prevent the progression of CKD. UPLC-QTOF/HDMS was employed for metabolic profiling of adenine-induced CKD and to investigate the nephroprotective effects of ergone. Pharmacology parameters including blood biochemistry, histopathological evaluation and Western blot analysis were performed concurrently. The UPLC-MS data were analyzed by partial least squares-discriminate analysis, correlation analysis, heatmap analysis and mapped to KEGG pathways. Blood and serum biochemistry were observed to be significantly different in the CKD group than in the control group. In conjunction with biochemistry, histopathology and protein expression results, identified metabolites indicated perturbations in fatty acid metabolism, purine metabolism and amino acid metabolism as changes associated with adenine-induced CKD and the interventions of ergone. Upregulated expression of TGF-β1, ED-1, CTGF, bFGF and collagen I was observed in the CKD group. However, downregulated expression of these proteins was observed after oral administration of ergone. These results suggest that expression changes in these proteins had implications for fatty acid metabolism, purine metabolism and amino acid metabolism in the development of CKD and that ergone treatment could delay the development of CKD by normalizing or blocking abnormal changes in biomarker metabolites and protein expression in the CKD group.

## Introduction

Normal kidney function is required for the clearance of endogenous metabolite and xenobiotic waste products from the body while maintaining the balance of ions, fluid and many small molecules. Chronic kidney disease (CKD) is an important public health problem with approximately 10% of this population progressing to end stage renal disease [1]. Accumulating toxins cause difficulty in controlling blood pressure, impairs renal function, and worsens prognosis in CKD patients [2], [3]. Serum creatinine (Scr) and glomerular filtration rate are often used as markers for CKD. Yet knowledge of the complex molecular defects and pathophysiological mechanisms causing CKD remain unclear as researchers are hindered by analytical methodologies that limited their focus to a single or relatively few high risk biomarkers at one time. Metabonomics approaches open the possibility to identify and quantify changes in many small-molecule metabolites in complex biological samples [4]–[6]. Metabolomic analysis of patient samples or animal models of CKD have been conducted by ^1^H nuclear magnetic resonance (^1^H NMR), liquid chromatography-mass spectrometry (LC-MS) and gas chromatography-mass spectrometry [7], [8]. Among the different LC-MS techniques, ultra performance liquid chromatography-quadrupole time-of-flight high-definition mass spectrometry (UPLC-QTOF/HDMS) is especially suited for large-scale untargeted metabonomics due to its enhanced reproducibility of retention time, selectivity and sensitivity [9]–[13].

Ergosta-4,6,8(14),22-tetraen-3-one (ergone) is widely distributed in medicinal fungi, lichen and plants such as *Polyporus umbellatus*, *Vietnamese Xylaria* and *Cordyceps sinensis* [14]–[16]. Ergone possesses cytotoxic activity, diuretic activity and nephroprotective effect [17]–[20]. Our previous study demonstrated that ergone could prevent progression of renal injury and subsequent renal fibrosis [21]. Pharmacokinetic studies indicated ergone was mainly excreted into the rat feces via bile instead of urine with approximately 57% of the loading dose present in the feces within 24 h [22]–[25]. Although therapeutic efficacy of ergone for CKD was demonstrated, the biochemical mechanism of its action was still not fully understood. Recent LC-MS-based serum and urinary metabonomics of chronic renal failure (CRF) rats have been reported and suggest that ergone can markedly influence the process of interstitial fibrosis, which might be due to the melioration of amino acid metabolism and lecithin metabolism [26], [27]. Adenine was a nitrogen heterocycles compound and uric acid was its final metabolite. Normally, adenine was efficiently salvaged by adenine phosphoribosyltransferase and blood and urine had very low level of adenine. If adenine was superfluous in mammalian metabolism, adenine would become a significant substrate for synthesis 2,8-dihydroxyadenine via an 8-hydroxyadenine intermediate by xanthine dehydrogenase [28]. The low solubility of 2,8-dihydroxyadenine would lead to precipitation in renal tubules and then this led to accumulation of blood urea nitrogen (BUN) and Scr [29]. Long-term feeding adenine to rats caused metabolic abnormalities similar to CKD clinical symptoms in humans. CKD in humans can be reproduced in the rodent animal including rats or mice by adenine; and adenine-induced CKD model can provide a special opportunity to study the CKD development and pathogenesis as well as effects of interventions that target disease progression due to the presence of metabolic abnormalities, declining renal function and chronic progressive tubulo-interstitial nephritis. In this study, UPLC-QTOF/HDMS were applied to investigate CKD pathological changes and the therapeutic effects of ergone. Partial least squares-discriminate analysis (PLS-DA), correlation analysis and heatmap analysis were performed for investigating the metabolic changes. This study provides new insights into the pathological changes that occur during the initiation and progression of CKD. This work may also offer an approach to evaluate therapeutic effects of anti-fibrogenic drugs and their mechanisms of action.

## Materials and Methods

### CKD model and drug administration

The experimental protocol was approved by the Institutional Animal Care and Use Committee of the Northwest University, Shaanxi. Adenine-induced CKD model was reproduced as described in detail previously [30]. Briefly, male Sprague-Dawley rats (obtained from Xi'an Jiaotong University) weighing 190 to 210 g were divided into control group, CKD group and CKD + Ergone group (n = 8/group). CKD group and CKD + Ergone group then were given 200 mg/kg body weight of adenine by oral gavage one time each day continuously for three weeks, to induce experimental CKD. The CKD + Ergone group was administered ergone (10 mg/kg) 3 h after each adenine dose. Body weight of each rat was measured daily. After 3 weeks, rats were anesthetized with 10% urethane, blood samples drawn and animals sacrificed. Kidneys were immediately excised, washed with physiological saline and stored at −80°C until further processing for histopathological analysis.

### Determination of body weight, kidney index and blood sample

After three weeks, the rats were housed individually in metabolic cages for 24 h urinary collection and the body weight was measured. The kidney weight index was calculated by kidney weight/body weight. BUN, Scr, cholesterol, triglyceride, uric acid, potassium, sodium, chloridion, phosphorus, calcium and creatine kinase were measured using an Olympus AU640 automatic analyzer and Easylyte plus Analyzer. Plasma aldosterone concentration was measured using an Aldosterone Kit (Shanghai Meilian Biological Technology Co., LTD., Shanghai, China) following the manufacturer's instructions. Blood parameters were determined by HF-3800 analyzer.

### Histopathological evaluation and western blot analysis

Kidneys were fixed in 10% buffered formalin, dehydrated with graded ethanol and embedded in paraffin for sectioning. Five µm paraffin sections were mounted on glass slides, rehydrated with distilled water, and stained with haematoxylin and eosin (HE) and picro-sirius red for light microscope examination. ED-1 and transforming growth factor-β1 (TGF-β1) (Santa Cruz Biotechnology Company) immunohistochemical staining was performed as described in detail previously [30]. Western blot analysis including TGF-β1, ED-1, CTGF, bFGF and collagen I proteins were performed as described in detail previously [31]. Blots were developed using Enhanced Chemiluminescence Reagents by following a procedure provided by the manufacturer (Amersham Pharmacia Biotech, USA).

### Sample preparation

Metabonomic samples were prepared as described in detail previously [30]. Briefly, frozen kidney tissue was homogenized with 0.5 mL acetonitrile followed by organic extraction with acetonitrile. Lyophilized extracts were resuspended in acetonitrile/water (4∶1) and cleared by centrifugation at 13,000 rpm for 10 min prior to UPLC-MS analysis.

### Chromatography and mass spectrometry

Each sample was injected onto a 2.1 mm×100 mm ACQUITY 1.8 µm HSS T3 column using an ACQUITY UPLC system (Waters Corporation, USA). The gradient mobile phase comprised of water (A) and acetonitrile (B), each containing 0.1% formic acid. Each sample was resolved for 8 min at a flow rate of 0.45 mL/min. This UPLC-MS method has been extensively used for metabolomics of bio-fluids or tissues; UPLC gradients conditions and MS parameters have been described in detail [30]. All the acquisition and analysis of data were controlled by Waters MassLynx v4.1 and MakerLynx software.

### Analytical method assessment and statistical analysis

The repeatability and precision were determined for assessment of the developed UPLC-MS method as described in detail previously [32]. Data Analysis was performed as described in detail previously [11]. Briefly, the acquired UPLC-MS data were imported into Markerlynx software for peak detection and alignment. Each data run was normalized to the summed total ion intensity per chromatogram, and the resultant data matrices were analyzed for PLS-DA with the EZinfo 2.0 software package. Ion peaks of MS were assigned by MS and MS/MS analyses or interpreted with available metabolic and biochemical databases including HMDB, Chemspider and KEGG. Biomarkers were identified from loading plots of PLS-DA, and the biomarkers were chosen according to their contribution to the variation and correlation within the data set. Correlation analysis and heatmap analysis of the identified metabolites were performed by Metaboanalyst analysis. Additional statistical analyses were performed using SPSS 11.0. Metabolite differences were considered significant when test *P* values were less than 0.05.

## Results and Discussion

### Basic physical parameters

Table 1 showed the results of body weight, urinary volume and kidney weight index among the study groups. Body weight was significantly decreased in the CKD group compared with the control group (*P*<0.01). However, compared with the CKD group, body weight was markedly increased in the CKD + Ergone group. Urinary volume was significantly increased in the CKD group compared with the control group (*P*<0.01), but this increase was substantially reduced in the CKD + Ergone group. Similarly, the weight index of the kidney was markedly increased in the CKD group (*P*<0.01). However, the weight index of the kidney was significantly decreased in the CKD + Ergone group.

**Table 1:** Physiologic parameters of animals.

Parameter	Control	CKD	CKD + Ergone
Body weight (g)	345±23	237±34^**^	286±30^##^
Urinary volume (mL)	15±4	46±11^**^	30±8^##^
Kidney weight index(g/g×100)	0.81±0.06	4.19±0.53^**^	3.54±0.48^#^
BUN (mmol/L)	6.8±1.7	36.1±5.5^**^	25.4±4.9^##^
Scr (µmol/L)	34.6±2.6	124.2±15.2^**^	82.4±19.5^##^
Serum cholesterol (mmol/L)	2.04±0.43	2.48±0.34^*^	2.11±0.32^#^
Serum triglyceride (mmol/L)	0.55±0.25	1.11±0.34^**^	0.85±0.14^##^
Serum uric acid (µmol/L)	129±18	174±21^**^	151±16^#^
Serum potassium (mmol/L)	5.11±0.25	5.85±0.42^**^	5.31±0.25^##^
Serum sodium (mmol/L)	140±1	141±2	142±1
Serum chloridion (mmol/L)	100±1	101±2	104±4
Serum phosphorus (mmol/L)	1.96±0.46	3.22±0.76^**^	2.88±0.28^##^
Serum calcium (mmol/L)	2.46±0.04	2.34±0.06^**^	2.41±0.05^#^
Serum creatine kinase (mmol/L)	1976±242	2280±279^*^	2121±257
Serum aldosterone (ng/mL)	31.6±5.6	95.6±9.2^**^	38.3±7.8^##^
White blood cell (10^9^/L)	12.8±2.1	21.2±3.6^**^	17.5±3.2^#^
Red blood cell (10^12^/L)	7.2±0.2	6.6±0.5^**^	7.1±0.4^#^
Hemoglobin (g/L)	162.2±7.1	148.2±7.8^**^	151.5±10

Results are expressed as the means ± standard deviation, *P<0.05, **P<0.01 compared with control group; ^#^ *P*<0.05, ^##^ *P*<0.01 compared with CKD group.

### Biochemical parameters

Data were summarized in Table 1. The levels of BUN, Scr, cholesterol, triglyceride, uric acid, potassium, phosphorus and creatine kinase were markedly increased in the CKD group compared with the control group, and all these increases were improved by treatment with ergone (*P*<0.05). The level of calcium was significantly decreased in the CKD group compared with the control group (*P*<0.05), and this decrease was improved by treatment with ergone. The levels of sodium and chloride ions did not significantly change in the CKD group compared with the control group. Aldosterone is a primary regulator of extracellular fluid volume and electrolyte balance including sodium and potassium. The level of aldosterone was significantly higher in the CKD group than in the control group (*P*<0.01), and the level of aldosterone was significantly decreased by treatment with ergone. These results demonstrated that the rat model exhibited typical pathologic features associated with CKD. Changes in the levels of BUN, Scr, cholesterol, triglyceride, uric acid, potassium, phosphorus, calcium, creatine kinase and aldosterone were significantly improved by treatment with ergone.

The data for blood parameters were given in Table 1. A remarkable increase in white blood cell and a remarkable decrease in red blood cell and hemoglobin were revealed in the blood sample of the CKD group compared with the control group (*P*<0.01). These blood parameters showed that adenine can cause symptoms of anemia. Ergone could decrease white blood cell and partially restore red blood cell although not to the normal levels. These results demonstrated overall that ergone treatment could prevent or broadly alleviate changes associated with CKD.

### Histological findings

**Figure 1:**
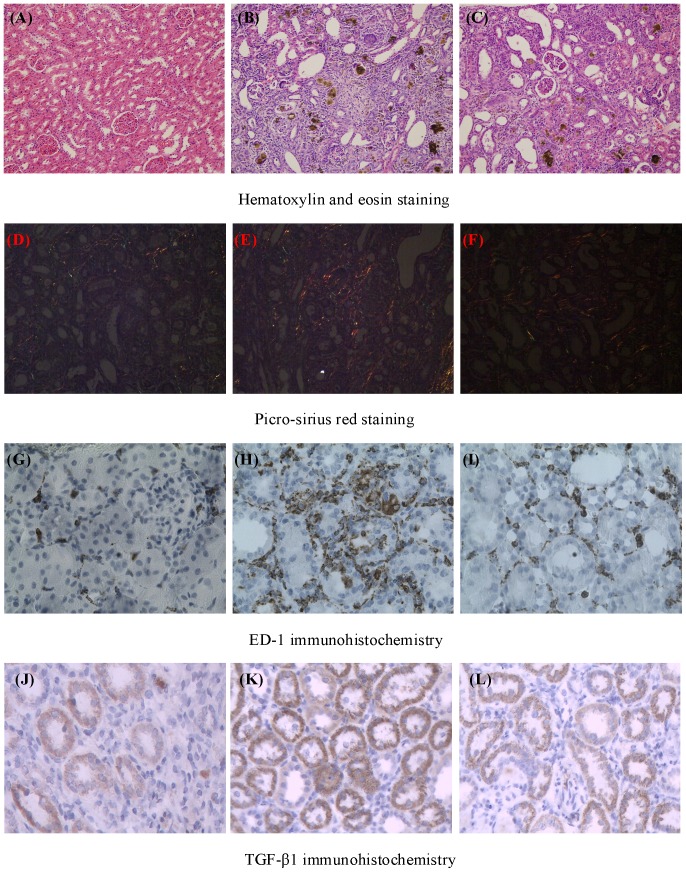
Histological findings of hematoxylin-eosin and picro-sirius red staining as well as ED-1 and TGF-β1 immunohistochemisrty. Histological findings of kidney hematoxylin-eosin staining from control (A), CKD (B) and CKD + Ergone groups (C) and picro-sirius red staining from control (D), CKD (E) and CKD + Ergone groups (F). Immunohistochemical findings by ED-1 antibody from the control (G), CKD (H) and CKD + Ergone group (I) and TGF-β1 antibody from the control (J), CKD (K) and CKD + Ergone group (L).

Fig. 1A to C showed the histological findings of kidney tissue HE staining from the control, CKD and CKD + Ergone groups. Both tubulo-interstitium and glomeruli of control group appeared normal, while CKD group had remarkable damage including severe inflammatory cell infiltration, marked tubular dilation and interstitial fibrosis. These changes were improved in the rats by treatment with ergone.

Picro-sirius red staining is a sensitive method for distinguishing type I and type III collagen fibers. Based on birefringence properties, in bright-field microscopy, collagen is red on a pale yellow background. Under polarized light, the larger collagen fibers (type I) are bright yellow or orange, and the thinner ones (type III), including reticular fibers, are green. Fig. 1D to F showed histologic findings of picro-sirius red staining in the control, CKD and CKD + Ergone groups. Expression of collagen I and collagen III proteins was significantly upregulated in the CKD group. However, protein expression was remarkable ameliorated in the CKD + Ergone group.

Macrophages play an important role in the development of tubulo-interstitial inflammation. We used ED-1 staining to reveal changes in macrophage infiltration in kidney tissues. The numbers of ED-1-positive cells were significantly increased in the interstitium of CKD group compared with the control group (Fig. 1G–H). The increase in ED-1-positive cells in the interstitium of the CKD group was remarkably suppressed by treatment with ergone (Fig. 1I).

Fig. 1J to L showed immunohistochemical staining for TGF-β1 from control, CKD and CKD + Ergone groups. Expression of TGF-β1 protein was significantly upregulated in the kidney tubule of CKD group. Compared with the CKD group, expression of TGF-β1 protein was significantly downregulated in the CKD + Ergone group. In conclusion, the above-mentioned results demonstrated that adenine-induced rats exhibited classical CKD pathological features. However, tubulo-interstitial injury was substantially ameliorated in the CKD + Ergone group.

### Multivariate analysis and biomarker identification

**Figure 2:**
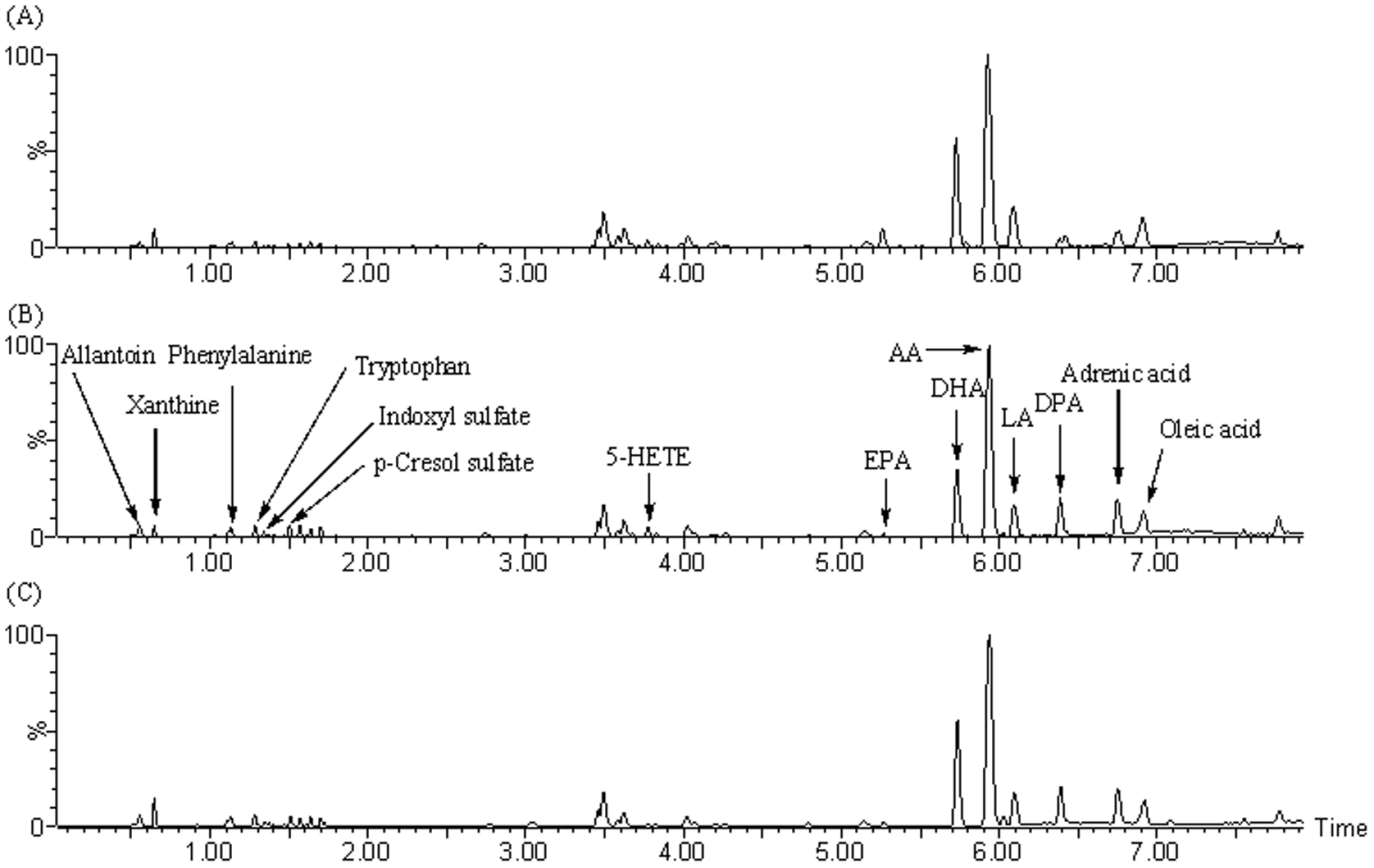
The UPLC-MS base peak intensity (BPI) chromatograms. (A) control group, (B) CKD group and (C) CKD + Ergone group. The chromatogram showed identified metabolites.

**Figure 3:**
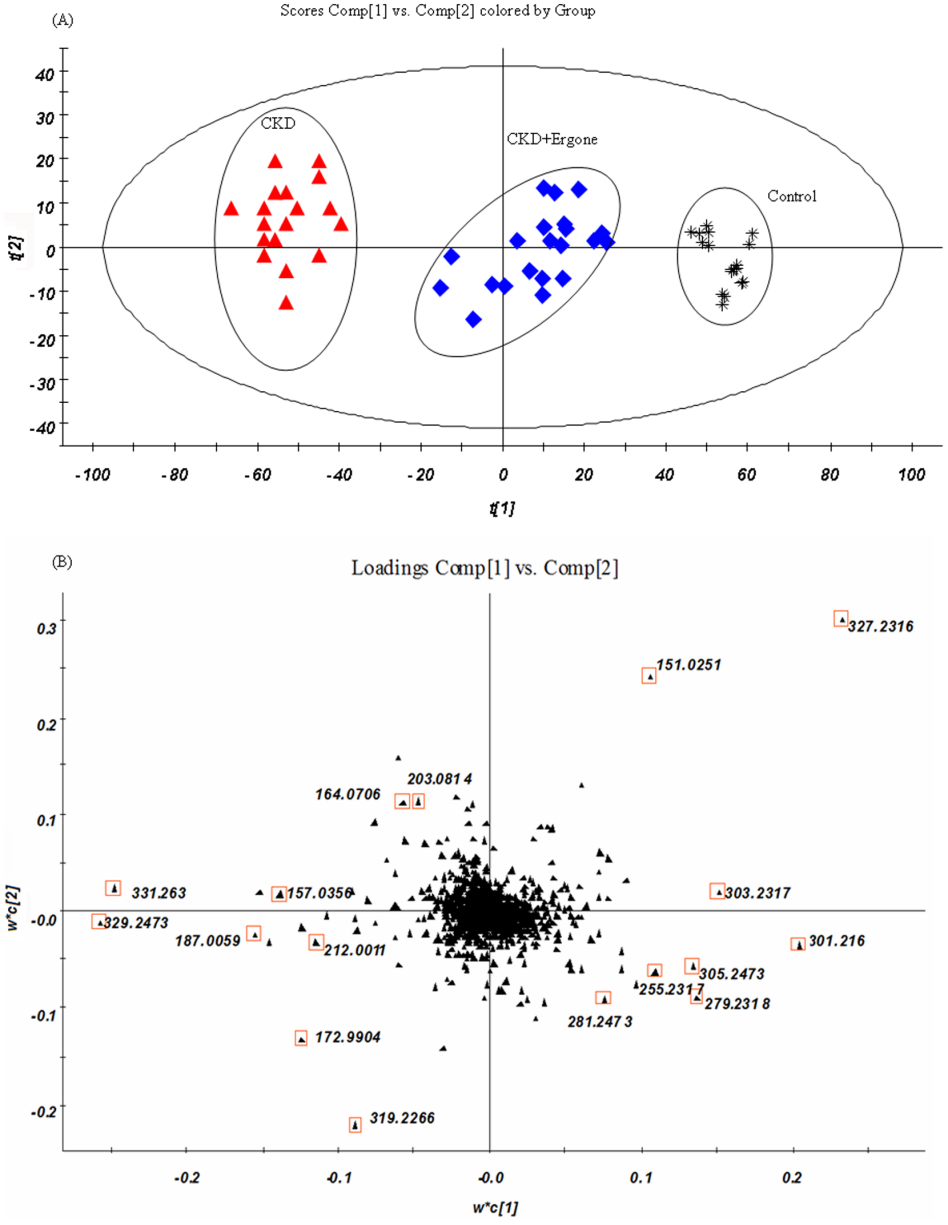
Partial least squares discriminant analysis of in the three different groups. PLS-DA scores plot (A) loading plot (B) of kidney tissue from (*) control, (▴) CKD and (♦) CKD + Ergone groups. The variables marked (□) are the metabolites selected as potential biomarkers.

Reproducibility of the UPLC-MS was determined from ten replicated analyses of the same kidney tissue sample interspersed throughout the analysis. The RSD of retention time and peak area are below 0.48% and 3.4%, respectively demonstrating good stability and reproducibility throughout the whole sequence of sample runs. Metabolic profiling of kidney tissue was acquired in negative ion mode (Fig. 2). PLS-DA analysis was performed on the result set of tissue biomarker concentrations. 2179 peaks were detected and processed by MarkerLynx *XS* using the treatment groups as category classifiers to obtain better discrimination among control, CKD and CKD + Ergone groups to reveal metabolic patterns significantly changed by treatment with ergone. The PLS-DA scores plot was shown in Fig. 3A. Corresponding loading plots (Fig. 3B) indicated candidate biomarkers (shown as retention time_m/z pairs) of 5.73_327.2316, 6.39_329.2473, 6.75_331.2630, 0.65_151.0251, 3.77_319.2266, 5.26_301.2160, 3.02_410.2356, 1.28_172.9904, 6.09_279.2318, 7.54_359.2941, 1.50_187.0059, 6.42_305.2473, 5.93_303.2317, 1.56_192.0655, 0.54_157.0356, 6.76_255.2317, 1.13_164.0706, 1.28_203.0814, 6.91_281.2473 and 1.34_212.0011. Seventeen metabolites were positively confirmed based on criteria established in a prior published study [30] and were shown in Table 2. Metabolites including linoleic acid (LA), docosapentaenoic acid (DPA), eicosatrienoic acid (ETA), indoxyl sulfate (IS), p-cresol sulfate (p-CS), allantoin, tryptophan and phenylalanine have also been reported by others [33]–[36]. Additional CKD-related biomarkers were discovered in the current study (Table 2).

**Table 2:** Identification of significantly differential endogenous metabolites in the rat kidney.

No.	*t*_R_ (min)	m/z	Formula	Metabolite	Trend^a^	Trend^b^	Related pathway
1	5.73	327.2316	C_22_H_32_O_2_	Docosahexaenoic acid	↓^***^	↑^###^	Fatty acid metabolism
2	6.39	329.2473	C_22_H_34_O_2_	Docosapentaenoic acid	↑^***^	↓^#^	Fatty acid metabolism
3	6.75	331.2630	C_22_H_36_O_2_	Adrenic acid	↑^***^	↓^#^	Fatty acid metabolism
4	3.77	319.2266	C_20_H_32_O_3_	5-Hydroxyeicosatetraenoic acid	↑^***^	↓^###^	Fatty acid metabolism
5	0.64	151.0251	C_5_H_4_N_4_O_2_	Xanthine	↓^***^	↑^###^	Purine metabolism
6	5.26	301.2160	C_20_H_30_O_2_	Eicosapentaenoic acid	↓^***^	↑^###^	Fatty acid metabolism
7	1.28	172.9904	C_7_HN_4_S	4-Hydroxybenzenesulfonic acid	↑^***^	↓^###^	Unknown metabolism
8	6.09	279.2318	C_18_H_32_O_2_	Linoleic acid	↓^***^	↓	Fatty acid metabolism
9	1.50	187.0059	C_7_H_8_O_4_S	p-Cresol sulfate	↑^***^	↓^###^	Phenylalanine metabolism
10	6.42	305.2473	C_20_H_34_O_2_	Eicosatrienoic acid	↓^***^	↓	Fatty acid metabolism
11	5.93	303.2317	C_20_H_32_O_2_	Arachidonic acid	↓^*^	↑	Fatty acid metabolism
12	0.53	157.0356	C_4_H_6_N_4_O_3_	Allantoin	↑^***^	↓^#^	Purine metabolism
13	6.76	255.2317	C_16_H_32_O_2_	Palmitic acid	↓^***^	↓	Fatty acid metabolism
14	1.13	164.0706	C_9_H_11_NO_2_	Phenylalanine	↑^**^	↑^##^	Phenylalanine metabolism
15	1.28	203.0814	C_11_H_12_N_2_O_2_	Tryptophan	↑^**^	↑^##^	Tryptophan metabolism
16	6.91	281.2473	C_18_H_34_O_2_	Oleic acid	↓^*^	↓	Fatty acid metabolism
17	1.34	212.0011	C_8_H_7_NO_4_S	Indoxyl sulfate	↑^***^	↓^###^	Tryptophan metabolism

a Change trend of CKD group vs control group.^ *^ *P*<0.05; ^**^ *P*<0.01; ^***^ *P*<0.001

b Change trend of CKD + Ergone group vs CKD group. ^#^ *P*<0.05; ^##^ *P*<0.01; ^###^ *P*<0.001.

The levels of potential biomarkers were labeled with (↓) downregulated and (↑) upregulated.

### The network of identified biomarkers and their functions

**Figure 4:**
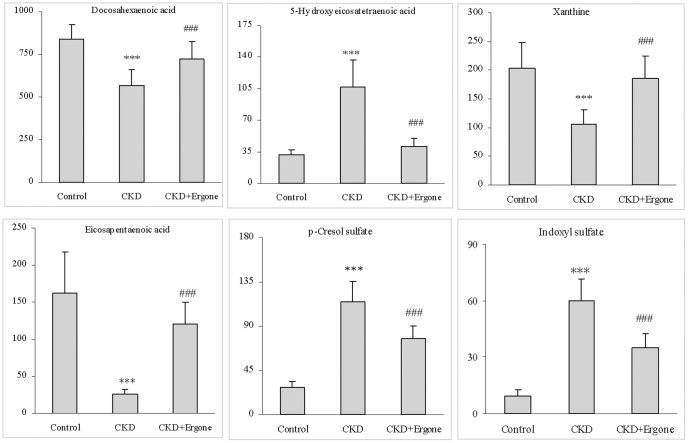
Change trend of identified biomarkers and Therapeutic effect of ergone. Six identified biomarkers completely reversed by treatment with ergone. *P<0.05, **P<0.01, ***P<0.001 significant difference compared with control group; ^#^ *P*<0.05, ^##^ *P*<0.01, ^###^ *P*<0.001 significant difference compared with CKD group.

**Figure 5:**
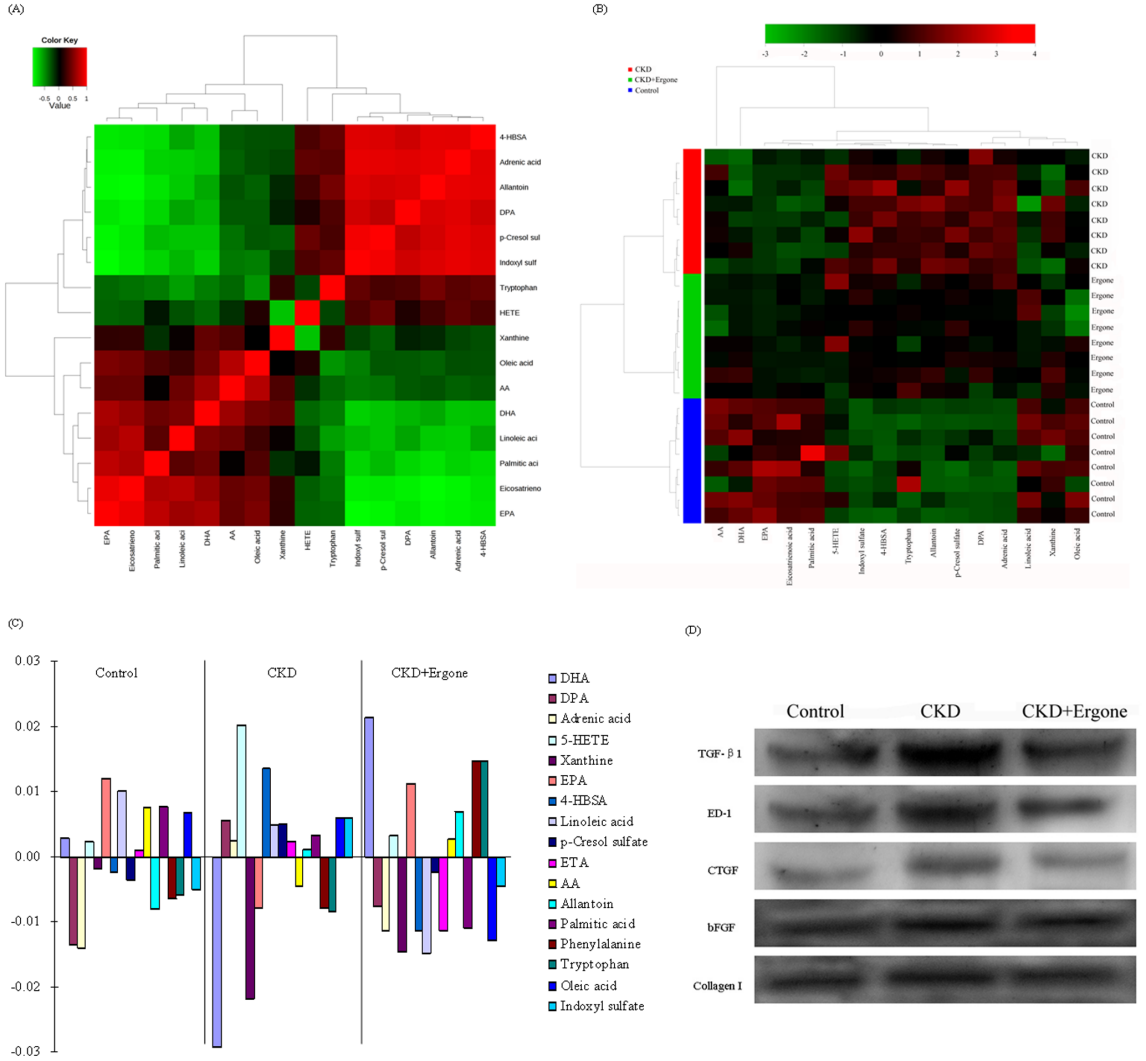
Multivariate statistical analysis and protein expression in the three different groups. Correlation analysis of the differential metabolites in the different groups (A). Heatmap for kidney of the identified metabolites in the different groups (B). The color of each section is proportional to the significance of change of metabolites (red, upregulated; green, downregulated). Rows: samples; Columns: metabolites. Correlation coefficient analysis among groups with corresponding markers in different groups (C). Variables are presented in control, CKD and CKD + Ergone groups. Values of correlations are shown in the vertical axis (low for negative correlations and upper for positive correlations) and corresponding metabolites represented to the right of the bars. Expression levels of TGF-β1, ED-1, CTGF, bFGF and Collagen I proteins were determined in the control, CKD and CKD + Ergone groups by Western blot analysis (D).

Significantly increased docosahexaenoic acid (DHA), xanthine, eicosapentaenoic acid (EPA), arachidonic acid (AA), phenylalanine and tryptophan were observed in the CKD + Ergone group compared with CKD group. However, significantly increased DPA, adrenic acid, 5-hydroxyeicosatetraenoic acid (5-HETE), 4-hydroxybenzenesulfonic acid (4-HBSA), LA, p-CS, ETA, allantoin, palmitic acid, oleic acid and IS were observed compared with CKD group (Table 2). The related metabolic pathways of identified biomarkers were shown in Table 2 by searching the KEGG PATHWAY Database. The 17 identified biomarkers were distributed in related pathways of fatty acid metabolism, purine metabolism and amino acid metabolism. Among those 17 identified biomarkers, DHA, 5-HETE, xanthine, EPA, IS and p-CS were completely reversed by treatment with ergone (Fig. 4). Fig. 5A presented correlation analysis of the differential metabolites that were shown in Table 2 and alignment showed the arithmetic mean of the relative intensity of identified metabolites in the control, CKD and CKD + Ergone groups. From the above plots, various metabolites could be identified as being associated with the separation in the control, CKD and CKD + Ergone groups.

To further better understand the metabolic differences among different groups, the metabolites were visualized in a clustering heatmap, which revealed directly the variation of each metabolite. Fig. 5B presented the heatmap built for all identified metabolites showing the relative increase (red) or decrease (green) compared with control group. The results indicated that the metabolic patterns of the biomarkers was significantly disturbed in the CKD group with the CKD + Ergone group located in between the CKD and control groups. The heatmap showed that ergone treatment exhibited preventive metabolic changes on CKD group by influencing multiple metabolic pathways. Specifically, significant increased in IS, 4-HBSA, allantoin, adrenic acid, p-CS and DPA were observed in CKD group but found to decrease again in the CKD + Ergone group. Similarly, significantly decreased DHA, AA, EPA and xanthine were observed in the CKD group but these changes were reversed in CKD + Ergone group. These results were summarized in Table 2 and Fig. 4. In addition, correlation coefficient analysis was used to investigate the relationship between identified metabolites and corresponding groups (Fig. 5C). The metabolites DHA, 5-HETE, EPA, LA, ETA, AA, palmitic acid and oleic acid were positively correlated with the control group and indicated an association with normal kidney function. The metabolites DPA, adrenic acid, 5-HETE, 4-HBSA, LA, p-CS, ETA, allantoin, palmitic acid, oleic acid and IS had a positive correlation with the CKD group and indicated a profiling chronic renal injury. The metabolites DHA, 5-HETE, EPA and AA have positive correlations with the CKD + Ergone group, while the metabolites including DPA, adrenic acid, xanthine, 4-HBSA, p-CS and IS are negatively correlated. These trends in metabolites changes were consistent with the protective effect by ergone on CKD group.

Progressive renal diseases including human renal diseases and animal models are the consequence of a process of destructive fibrosis. Typical characteristic of interstitial fibrosis is excess deposition of extracellular matrix components (TGF-β1, CTGF, bFGF, etc.), accumulated collagen proteins (collagens I, III, V, VII, XV, fibronectin) and associated glycoproteins. Many investigators have focused principally on the molecular pathogenesis of interstitial fibrosis owing to the correlation between the level of interstitial fibrosis and kidney functional injury. TGF-β was a central mediator of renal fibrosis and TGF-β1 has been most extensively investigated in renal fibrosis. TGF-β1 induced expression of CTGF, and CTGF can in turn enhance TGF-β1 signaling, along with a number of other pro-fibrotic factors including ED-1, vascular endothelial growth factor and insulin-like growth factor-1. bFGF stimulates release of preformed latent TGF-β1 from proximal tubular cells and bFGF expression also increases in tubular and/or interstitial cell. To study the relation between identified biomarkers and proteins, the expression of TGF-β1, ED-1, CTGF, bFGF and collagen I proteins was evaluated by Western blotting method. Upregulated expression of TGF-β1, ED-1, CTGF, bFGF and collagen I was observed in the CKD group compared with control group (Fig. 5D). However, amelioration of expression of these proteins was observed after oral administration of ergone.

In the current study, it was found that polyunsaturated fatty acids were the most important CKD-related metabolites and ten polyunsaturated fatty acids accounts for 60% of all the identified metabolites. Polyunsaturated fatty acids were the major components of cytoplasmic membrane and they were related to atherosclerotic and inflammatory diseases [37]. DHA, 5-HETE and EPA were reversed completely by treatment with ergone (Fig. 4). Beneficial effects of n-3 polyunsaturated fatty acids including DHA and EPA were observed in animal models and human nephropathies. Levels of DHA, EPA and LA were remarkably lower in hemodialysis patients than in CKD patients [38]. These lipids can impact the synthesis of inflammatory factors by the regulation in balance of n-3-derived eicosanoid and n-6-derived eicosanoid and the direct action on endothelium function and on major cytokine mediators of inflammation [39], [40]. Reports have demonstrated lower lipid peroxidation of n-3 fatty acids that modulate oxidative responses in subjects exposed to stress [41], [42]. This may be associated with the assembly of n-3 fatty acid into lipoproteins and the reduced opportunity for free radical attack of double bonds, inhibition of phospholipase A2 (pro-oxidant enzyme) and stimulation of antioxidant enzymes [43]. N-3 fatty acids were shown to act both by replacing eicosanoid substrate AA and inhibiting AA metabolism and by altering inflammatory gene expression through transcription factor activation [44], [45]. 5-HETE is a metabolite of AA metabolized by P450 enzymes. Interestingly, the level of 5-HETE was reversed closer to control level in the CKD + Ergone group. Addition of AA to mesangial cells could induce upregulation of TGF-β1, CTGF, fibronectin and collagen IV expression, while EPA and DHA had no stimulatory effects on mesangial cells. On the contrary, the co-exposure of cells to EPA and DHA could suppress the AA-induced upregulation of TGF-β1, fibronectin, CTGF and collagen IV expression [46], which were consistent with our protein expression results (Fig. 5D).

Uremic toxins including IS and p-CS contributed to the pathological process of CKD. A previous study demonstrated a significant association between serum IS and p-CS levels and CKD progression [47]. Accumulating evidence has demonstrated that IS and p-CS had important effects on chronic kidney injury [48], [49]. Increased renal IS and p-CS were observed in the CKD group compared with control group and a beneficial decreased renal IS and p-CS were revealed in the CKD + Ergone group. TGF-β1 was recognized as both a fibrogenic and inflammatory cytokine and played a critical role in kidney injury. It was reported that IS could upregulate TGF-β1 expression in uremic kidney, which enhances the renal expression of tissue inhibitor of metalloproteinase-1 and collagen I, leading to CKD progression [50]. Another study showed that decreased IS with uremic toxin binders could significantly downregulate TGF-β1 expression [51]. The current results demonstrated that ergone could downregulate TGF-β1 and collagen I protein expression by promoting decreases of IS and p-CS in the CKD group (Fig. 5D).

Amino acids were substrates for metabolic energy, protein synthesis, gluconeogenesis and ketogenesis. Increased renal phenylalanine and tryptophan were observed in the CKD + Ergone group compared with the CKD group (Table 2). A major metabolic pathway of phenylalanine is its hydroxylation by phenylalanine hydroxylase to tyrosine. It was reported that decreased phenylalanine was observed in kidney medullar tissue, plasma and urine of adenine-induced CKD rats compared with control rats [52]–[54]. Also consistent with this observation was the finding that phenylalanine was higher in CKD patients than in healthy subjects [55]. A separate ^1^H NMR metabonomics showed increased serum phenylalanine was observed in both low-risk immunoglobulin A nephropathy patients and high-risk patients with nephropathies [56]. Tryptophan was either incorporated into proteins or broken down for energy and metabolic intermediates. The previous report showed decreased serum and increased urinary tryptophan was observed in adenine-induced CKD rats [33], [53]. The UPLC-MS metabolomics study showed decreased serum tryptophan in CKD patients [55]. These cited data demonstrated that the metabolic pathways for phenylalanine and tryptophan may be partly disturbed in the CKD group.

## Conclusion

We have applied a pharmaco-metabolomics approach to the study of the pathology of adenine-induced CKD, as well as the related biochemical mechanisms after treatment with ergone. Blood biochemistry parameters were significantly altered in the CKD group compared with the control group. Multiple metabolites were correlated with this progressive chronic renal injury in the CKD group. Expression of TGF-β1, ED-1, CTGF, bFGF and collagen I was upregulated in the CKD group while expression of these proteins was reversed after oral administration of ergone. Hence, the combination of blood biochemistry, histopathology, protein expression and metabonomics data indicated significant perturbations of fatty acid metabolism, purine metabolism and amino acid metabolism related to adenine-induced CKD and sensitive to the intervention with ergone. Our results showed that expression of these proteins was likely to have a bearing on fatty acid metabolism, purine metabolism and amino acid metabolism in the development of CKD and ergone treatment could delay or reverse this development and/or block the disorder in key metabolites associated with adenine-induced CKD.
